# Targeted Knockout of MDA5 and TLR3 in the DF-1 Chicken Fibroblast Cell Line Impairs Innate Immune Response Against RNA Ligands

**DOI:** 10.3389/fimmu.2020.00678

**Published:** 2020-04-30

**Authors:** Su Bin Lee, Young Hyun Park, Kelly Chungu, Seung Je Woo, Soo Taek Han, Hee Jung Choi, Deivendran Rengaraj, Jae Yong Han

**Affiliations:** Department of Agricultural Biotechnology and Research Institute of Agriculture and Life Sciences, College of Agriculture and Life Sciences, Seoul National University, Seoul, South Korea

**Keywords:** chicken, CRISPR/Cas9, innate immunity, MDA5, PRRs, RNA ligand, TLRs

## Abstract

The innate immune system, which senses invading pathogens, plays a critical role as the first line of host defense. After recognition of foreign RNA ligands (e.g., RNA viruses), host cells generate an innate immune or antiviral response *via* the interferon-mediated signaling pathway. Retinoic acid-inducible gene I (RIG-1) acts as a major sensor that recognizes a broad range of RNA ligands in mammals; however, chickens lack a RIG-1 homolog, meaning that RNA ligands should be recognized by other cellular sensors such as melanoma differentiation-associated protein 5 (MDA5) and toll-like receptors (TLRs). However, it is unclear which of these cellular sensors compensates for the loss of RIG-1 to act as the major sensor for RNA ligands. Here, we show that chicken MDA5 (cMDA5), rather than chicken TLRs (cTLRs), plays a pivotal role in the recognition of RNA ligands, including poly I:C and influenza virus. First, we used a knockdown approach to show that both cMDA5 and cTLR3 play roles in inducing interferon-mediated innate immune responses against RNA ligands in chicken DF-1 cells. Furthermore, targeted knockout of cMDA5 or cTLR3 in chicken DF-1 cells revealed that loss of cMDA5 impaired the innate immune responses against RNA ligands; however, the responses against RNA ligands were retained after loss of cTLR3. In addition, double knockout of cMDA5 and cTLR3 in chicken DF-1 cells abolished the innate immune responses against RNA ligands, suggesting that cMDA5 is the major sensor whereas cTLR3 is a secondary sensor. Taken together, these findings provide an understanding of the functional role of cMDA5 in the recognition of RNA ligands in chicken DF-1 cells and may facilitate the development of an innate immune-deficient cell line or chicken model.

## Introduction

Host pattern recognition receptors (PRRs), which are part of the innate immune system, form the first line of defense against invading pathogens. These receptors recognize pathogen-associated molecular patterns (PAMPs) and trigger interferon-mediated innate immune responses (1–3).

Upon recognition of invading pathogens, PRRs form complexes with adaptor proteins such as the mitochondrial antiviral signaling (MAVS) protein, TIR domain-containing adaptor-inducing interferon-β (TRIF), and myeloid differentiation primary response 88 (Myd88) (4–6). Subsequently, these PRR complexes activate interferon regulatory factor 3/7 (IRF3/IRF7) and the nuclear factor kappa-light-chain-enhancer of activated B cells (NF-κB), which in turn activate the interferon-mediated signaling pathways and induce the expression of interferon-stimulated genes (1, 7–10).

In mammalian species, retinoic acid-inducible gene I (RIG-1), melanoma differentiation-associated protein 5 (MDA5), DExH-box helicase 58 (DHX58), Toll-like receptors (TLRs), and nucleotide-binding oligomerization domain-containing (NOD)-like receptors (NLRs) act as PRRs that recognize RNA viruses. Among these, RIG-I and MDA5, known as the RIG-I-like receptors (RLRs), are the major PRRs for RNA virus recognition (1, 11, 12). RIG-1 recognizes a broad range of RNA viruses harboring 5′ triphosphate (5′ppp) moieties, including influenza virus and Sendai virus (5, 12–14), whereas MDA5 recognizes double-stranded RNA (dsRNA) patterns, including poly I:C, picornaviruses, and encephalomyocarditis virus (EMCV) (15, 16). Both RIG-1 and MDA5 contain N-terminal caspase activation and recruitment domains (CARDs), which activate downstream signaling pathways by interacting with the MAVS protein after virus recognition (17, 18). It is notable that RIG-1 and MDA5 can be regulated negatively or positively by DHX58 receptors, which lack CARDs (19, 20).

In addition to the RLR family, the TLR family recognizes a wide range of PAMPs, including bacteria, viruses, and other diverse pathogens (21–24). In mammals, TLR3 and TLR7/8, both of which are located in endosomes, recognize dsRNA ligand-like poly I:C and single-stranded RNA (ssRNA) ligand-like resiquimod (R-848), respectively (25–28). Furthermore, TLR3 and TLR7/8 recognize RNA viruses such as influenza virus, thereby inducing type I IFN-mediated signaling pathways (5, 28–30).

Within avian species, ducks harbor the RIG-1 gene; however, chickens lack the RIG-1 gene due to loss of a RIG-1 homolog prior to domestication, suggesting that chickens are more susceptible to infection by RNA viruses than ducks (31, 32). Despite the absence of RIG-1, the IFN-mediated signaling pathway in chickens is activated by chicken MDA5 (cMDA5) in response to the influenza virus (33). Furthermore, cMDA5 partially compensates for the absence of RIG-1 by cooperating with CARD adapter-inducing interferon beta (CARDIF) and DHX58; this allows recognition of the influenza virus and subsequent activation of the IFN signaling pathway (11, 34). Intriguingly, the tree shrew (a small mammal lacking RIG-1) expresses MDA5 (tMDA5), which recognizes 5′ppp RNA and Sendai virus and activates the IFN signaling pathway; this suggests that tMDA5 can (at least partially) compensate for the loss of RIG-1 function (34). In this regard, cMDA5 may be responsible for the recognition of RNA ligands in a manner similar to tMDA5. However, the specific underlying mechanisms have not been identified through functional studies.

Here, we performed small interfering RNA (siRNA)-mediated gene silencing experiments in chicken DF-1 cells to determine which PRRs (including RLR and TLR family members) sense RNA ligands. In addition, we used the clustered regularly interspaced short palindromic repeats (CRISPR)/CRISPR-associated protein 9 (CRISPR/Cas9) system to establish cMDA5-targeted knockout (KO) DF-1 clones and then used them to examine the functional role of cMDA5 in the chicken innate immune system. Furthermore, we knocked out cTLR3 in DF-1 cells and analyzed the functional role of these PRRs in response to RNA ligand PAMP mimics and influenza virus to ascertain whether cMDA5 or cTLR3 acts as a pivotal sensor for the recognition of RNA ligands.

## Materials and Methods

### Construction of CRISPR/Cas9 Vectors

The CRISPR/Cas9 vectors targeting the cMDA5 and cTLR3 genes were constructed using the pX459 vector, as previously reported (35). To insert the guide RNA (gRNA) sequences into the CRISPR/Cas9 vectors, sense and antisense oligonucleotides were synthesized (Bionics, Seoul, South Korea) (Supplementary Table S1) and annealed using the following thermocycling conditions: 30 s at 95°C, 2 min at 72°C, 2 min at 37°C, and 2 min at 25°C. The annealed oligonucleotides for each gRNA were ligated into the pX459 vector using the Golden Gate assembly method, and the constructed CRISPR/Cas9 vectors were validated by Sanger sequencing.

### Cell Culture, Transfection, and Clonal Selection of DF-1 Cells

Chicken DF-1 fibroblast cells (CRL-12203; ATCC, Manassas, VA, United States) were maintained in Dulbecco’s minimum essential medium (DMEM; Hyclone, Logan, UT, United States), supplemented with 10% fetal bovine serum (Hyclone) and 1× antibiotic-antimycotic (ABAM; Thermo Fisher-Invitrogen, Santa Clara, CA, United States). Cells were cultured at 37°C in an incubator with an atmosphere of 5% CO_2_ and a relative humidity of 60–70%. For transfection, 2 μg of cMDA5 #1, cMDA5 #2, or cTLR3 #1 was mixed with 2 μl of Lipofectamine 2000 reagent (Thermo Fisher-Invitrogen) in Opti-MEM (Thermo Fisher-Invitrogen). This mixture was then applied to 2 × 10^5^ DF-1 cells in 12-well culture plates. Approximately 24 h later, puromycin (1 μg/ml; GIBCO Invitrogen, New York, NY, United States) was added to the culture medium to select transfected DF-1 cells. A complete selection period required 3–4 days. Single puromycin-selected DF-1 cells were seeded into individual wells of a 96-well plate containing the culture medium. After clonal expansion, genomic DNA was extracted for sequencing analysis.

### T7E1 Assay and Genomic DNA Sequencing Analysis

To evaluate the targeting efficiency of the transfected CRISPR/Cas9 vectors in chicken DF-1 cells, genomic DNA was extracted following puromycin selection. Genomic regions encompassing the CRISPR/Cas9 target sites were amplified using specific primer sets (Supplementary Table S1). Following denaturation, the amplicons were reannealed to form heteroduplex DNA. Subsequently, the heteroduplex amplicons were treated with T7 endonuclease I (T7E1; NEB, United States) for 20 min at 37°C and then analyzed by 1% agarose gel electrophoresis. For sequence analysis, PCR products containing the target site were cloned into the pGEM-T Easy vector (Promega, Fitchburg, WI, United States) and sequenced using an ABI Prism 3730 XL DNA Analyzer (Thermo Fisher-Applied Biosystems, Carlsbad, CA, United States). The sequences were analyzed against the assembled chicken genomes using BLAST^1^ and Geneious R6 software (Biomatters Ltd., Auckland, New Zealand).

### Viruses

Reverse genetics systems were used to generate low pathogenic avian influenza virus (AIV; PR8-H5N8 strain) from eight bidirectional PHW2000 plasmids encoding the PB1, PB2, PA, HA, NA, NP, NS, and M genes, due to its convenience to modify viral genome by simple substitution (11, 36, 37). Viruses were rescued by co-transfection of the eight bidirectional plasmids into co-cultured Madin–Darby canine kidney cells (MDCK; ATCC, CCL-34) and human 293T embryonic kidney cells (HEK293T; ATCC, CRL-11268). The generated viruses were grown in MDCK infection medium comprising DMEM supplemented with 0.3% bovine serum albumin (BSA), 1 × ABAM, and 1 μg/ml TPCK-treated trypsin (Sigma-Aldrich, St. Louis, MO, United States) and then incubated at 37°C for 48 h. The virus stocks were further propagated in 10-day-old embryonated chicken eggs. Aliquots of the infectious virus were stored at −80°C for further experiments. All work with low-pathogenicity viruses was conducted in a biosafety level 2 facility approved by the Institutional Biosafety Committee, Seoul National University.

### Viral Titration of Infected Cells

Titration of infected cells was performed in MDCK cells to determine the median tissue culture infectious dose (TCID_50_). In brief, supernatants of the infected cells were used to infect confluent layers of MDCK cells in 96-well plates containing serum-free DMEM supplemented with 0.3% BSA, 1% penicillin/streptomycin, and 1 μg/ml TPCK-trypsin. Serial dilutions of the supernatant were added to five wells of a 96-well culture plate in triplicate. After 72–96 h, the cytopathic effects (CPEs) were examined by observing the detached cells through an inverted microscope and quantified by crystal violet (Sigma-Aldrich) staining. The TCID_50_ values per milliliter were calculated using the Spearman–Karber formula (38).

### siRNA-Mediated Gene Knockdown

DF-1 cells were seeded into the wells of a 12-well plate (each of which contained 1 ml medium) at a density of 2 × 10^5^ cells per well. Next, the cells were transfected with siRNAs targeting cMDA5, cTLR3, and cTLR7 (20 pmol/ml) using RNAiMAX (Invitrogen). The siRNAs were designed and synthesized by Bioneer Corporation, Daejeon, South Korea (Supplementary Table S2). Controls comprised siRNA with sequences non-complementary to NC-siRNA in the chicken genome. At 48 h of siRNA transfection, the knockdown efficiency of the selected genes and their effects on transcription were measured by quantitative reverse-transcription PCR (RT-qPCR).

### Analysis of Gene Expression Using RT-qPCR

Total RNA was extracted from the test samples using an RNeasy mini kit (Qiagen, Hilden, Germany) and then reverse-transcribed using the Superscript IV First-Strand Synthesis System (Thermo Fisher-Invitrogen). RT-qPCR was performed using a StepOnePlus real-time PCR system (Applied Biosystems, Foster City, CA, United States). The PCR reaction mixture contained 2 μl PCR buffer, 1 μl of 20 × EvaGreen qPCR dye (Biotium, Hayward, CA, United States), 0.5 μl of 10 mM dNTP mixture, 10 pmol each of gene-specific forward and reverse primers (Supplementary Table S1), 1 μl cDNA, and 1 U Taq DNA polymerase (final volume, 20 μl). RT-qPCR was performed in triplicate. Relative quantification of the target gene expression in infected cells was performed.

### Stimulation of Cell Lines With PAMPs

To investigate IFN signaling, DF-1 cell lines and controls were exposed to 1.5 μg/ml poly I:C-HMW/LyoVec (an average size of 1.5–8 kb; Cat# tlrl-piclv, Invivogen, San Diego, CA, United States), a complex between HMW poly I:C and the transfection reagent LyoVec, or a solvent (mock; control). Cells were collected at 24 h post-stimulation for RNA extraction following the manufacturer’s instructions. Three biological replicates were used per group.

### Luciferase Reporter Assays

To assess IFN-β promoter activity, 1 × 10^5^ DF-1 cells were seeded in 24-well plates and cultured overnight prior to transfection with 400 ng cMDA5 expression plasmid/empty vector, 200 ng firefly reporter plasmid (cIFN-β-Luc), and 20 ng pGL-4.53 plasmid expressing Renilla luciferase (used as the internal reference). The dual-luciferase reporter assays were performed according to the manufacturer’s instructions (Promega). After transfection for 24 h, the cells were stimulated with poly I:C (1.5 μg/ml) for 24 h. The cells were lysed and the samples were assayed for firefly and Renilla luciferase activity using the Dual-Luciferase Reporter Assay System (Promega). Promoter activity was normalized to the Renilla luciferase activity. All reporter assays were repeated at least three times.

### Statistical Analysis

Statistical analysis was performed using GraphPad Prism software (GraphPad Software, San Diego, CA, United States). Significant differences between groups were determined by Student’s *t*-tests, one-way or two-way analysis of variance (ANOVA) with Bonferroni’s multiple comparison. A *P*-value < 0.05 was deemed significant.

## Results

### cMDA5 Is a Potent Sensor of RNA Ligands in Chicken Cells, Whereas cTLR3 Plays a Secondary Role

**FIGURE 1:**
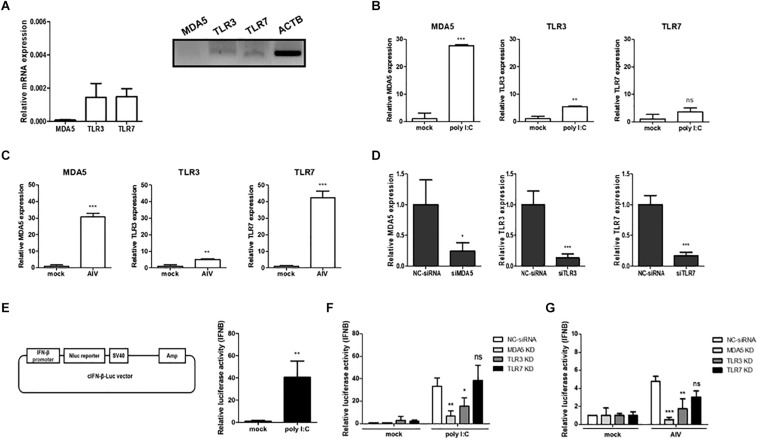
Contribution of MDA5, TLR3, and TLR7 to sensing RNA ligands in chicken cells. **(A)** The relative expression levels of MDA5, TLR3, and TLR7 mRNAs were measured by quantitative reverse-transcription PCR (RT-qPCR) and RT-PCR in the prepared cDNA from DF-1 cells. **(B)** DF-1 cells were stimulated for 24 h with poly I:C (1.5 μg/ml) and then harvested. The relative expressions of MDA5, TLR3, and TLR7 mRNAs were measured by RT-qPCR. **(C)** DF-1 cells were infected with avian influenza virus (AIV; MOI = 0.01) for 3 h and then harvested. The relative expressions of MDA5, TLR3, and TLR7 mRNAs were measured by RT-qPCR. **(D)** DF-1 cells were transfected for 48 h with siRNA targeting MDA5, TLR3, and TLR7 (20 pM). Non-complementary sequences were used as a siRNA control. After transfection, the cells were harvested and knockdown efficiency was analyzed by RT-qPCR. **(E)** Vector map of cIFN-β-Luc used for the luciferase reporter assay. DF-1 cells were co-transfected with the cIFN-β-Luc vector (200 ng) and pGL4.53 (20 ng) and then stimulated for 24 h with poly I:C. IFN-β promoter activity was analyzed with a luciferase assay. **(F)** Wild-type (WT) DF-1 cells were transfected with siRNA targeting MDA5, TLR3, and TLR7 (20 pM). After 48 h, the cells were co-transfected with cIFN-β-Luc vector (200 ng) and pGL4.53 (20 ng) and then stimulated for 24 h with poly I:C. After stimulation, the cells were harvested for a luciferase assay. **(G)** WT DF-1 cells were transfected with siRNA targeting MDA5, TLR3, and TLR7 (20 pM). After 48 h, the cells were co-transfected with IFN-β-Luc vector (200 ng) and pGL4.53 (20 ng) and then infected with AIV (MOI = 1) for 8 h. Relative IFN-β promoter activity was measured with a luciferase assay. The data are represented as the mean ± standard deviation (*n* = 3). Students *t*-test was used for comparing the significant difference between groups in **(A–E)**. One-way ANOVA with Bonferroni’s multiple comparison was used for comparing the significant difference between poly I:C or AIV groups in **(F,G)**. *P < 0.05, **P < 0.01, and ***P < 0.001. *ns*, non-significant.

To better understand the pivotal roles played by cMDA5, cTLR3, and cTLR7 in the absence of RIG-1, we first examined the expressions of mRNAs encoding cMDA5, cTLR3, and cTLR7 in chicken DF-1 cells. Without any stimulation, cMDA5, cTLR3, and cTLR7 showed low expression levels compared to ACTB in DF-1 cells (Figure 1A). After exposure to poly I:C, the expressions of cMDA5 and cTLR3 mRNAs, but not cTLR7 mRNA, were significantly upregulated (Figure 1B). We also exposed DF-1 cells to AIV [PR8-H5N8 strain, multiplicity of infection (MOI) = 0.01] and examined the expressions of cMDA5, cTLR3, and cTLR7 in infected cells. Unlike stimulation with poly I:C, we found that all three genes were upregulated in AIV-infected DF-1 cells (Figure 1C).

Next, we performed siRNA-meditated gene silencing of cMDA5, cTLR3, and cTLR7 to determine their functional roles in IFN-mediated innate immunity. We transfected DF-1 cells with gene-specific siRNA or NC-siRNA and analyzed the knockdown efficiency by RT-qPCR at 48 h post-transfection. The results showed that, on average, the expressions of cMDA5, cTLR3, and cTLR7 mRNAs were silenced by 76, 87, and 84%, respectively (Figure 1D). Next, to investigate interferon-mediated antiviral activity, we constructed a vector harboring a chicken IFN-β promoter luciferase reporter and performed a luciferase assay to reveal a significant increase in IFN-β promoter activity after stimulation with poly I:C compared with mock (Figure 1E).

Finally, we examined the effect of gene knockdown on IFN-β promoter activity after stimulation with poly I:C or AIV. Compared with NC-siRNA, the knockdown of cMDA5 and cTLR3 resulted in a significant reduction of IFN-β promoter activity in DF-1 cells after poly I:C stimulation, whereas the knockdown of cTLR7 did not affect IFN-β promoter activity (Figure 1F). Furthermore, we infected cMDA5-, cTLR3-, or cTLR7-silenced DF-1 cells with AIV (MOI = 1.0) and analyzed IFN-β promoter activity. The results showed that silencing of all genes in AIV-infected cells reduced IFN-β promoter activity. The IFN-β promoter activity fell by about 10-fold, 3-fold, and 1.8-fold after silencing of cMDA5, cTLR3, and cTLR7, respectively (Figure 1G).

### Establishment of cMDA5 Knockout DF-1 Clones Using CRISPR/Cas9-Mediated Genome Editing

**FIGURE 2:**
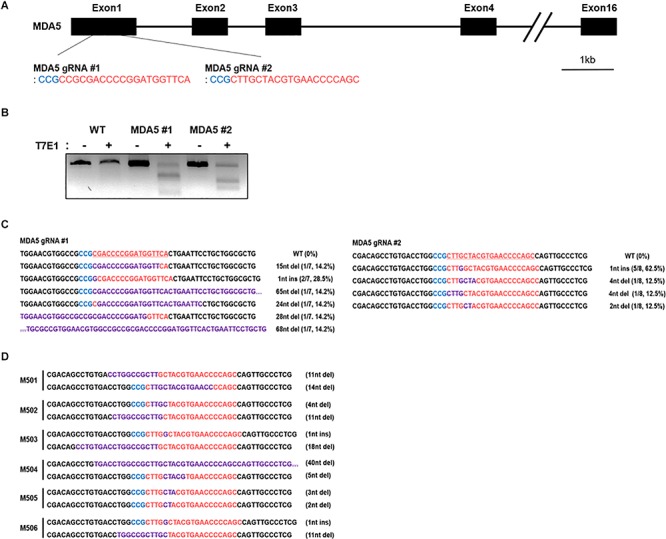
Establishment of chicken MDA5 (cMDA5) knockout cell lines using the CRISPR/Cas9 system. **(A)** Structure of the cMDA5 gene and location of the gRNA #1 and gRNA #2 sites. **(B)** T7E1 assays were performed in DF-1 cells transfected with the cMDA5 gRNA #1 and gRNA#2 CRISPR/Cas9 vectors. Wild-type DF-1 cells were used as the control. **(C)** Sequencing analysis of the transfected DF-1 cells. **(D)** Sequencing analysis of cMDA5 single-knockout cells after clonal expansion. The *red letters* indicate guide RNA binding sites, the *blue letters* indicate protospacer adjacent motif (*PAM*) sequences, and *purple letters* indicate mutation patterns. *M5* denotes MDA5 knockout single cell clones.

To knock out cMDA5 in DF-1 cells completely, we constructed two CRISPR/Cas9 vectors (containing MDA5 #1 gRNA and MDA5 #2 gRNA, respectively) targeting the first exon of cMDA5 to introduce an in-del mutation-mediated premature stop codon (Figure 2A). DF-1 cells were transfected with each CRISPR/Cas9 vector and then subjected to selection with puromycin. The results of the T7E1 assays showed that DF-1 cells transfected with the CRISPR/Cas9 vector harbored in-del mutations at the target locus (Figure 2B). To analyze the mutation efficiency, we sequenced the target region of the transfected cells. The results showed that the mutation rate in cMDA5 #1 and cMDA5 #2 was 100% (7/7 and 8/8, respectively) (Figure 2C). Next, we established individual DF-1 clones from cM#1 (cMDA5 #1) vector-transfected DF-1 cells. After DNA sequencing analysis, we identified six cMDA5 KO clones (M501–M506) harboring frameshift mutations within the first exon of cMDA5, resulting in a premature stop codon (Figure 2D). We used M502 DF-1 as a representative cMDA5 KO clone for subsequent experiments.

### Effect of cMDA5 KO on the Replication of Influenza Virus and IFN-Mediated Antiviral Activity in Chicken Cells

**FIGURE 3:**
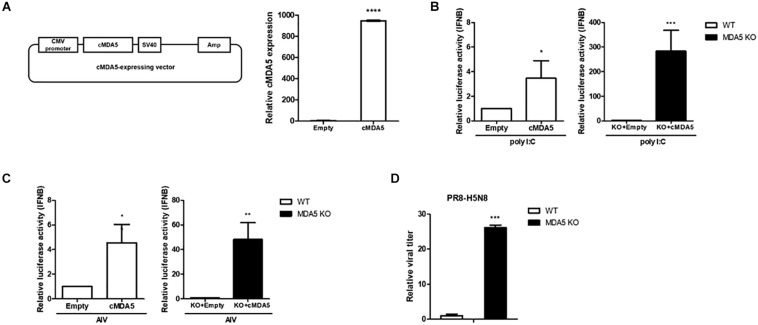
Effects of cMDA5 on virus replication and the type I IFN pathway. **(A)** Vector map of the cytomegalovirus (CMV)-driven cMDA5-expressing plasmid. Wild-type (WT) DF-1 cells were transfected with the cMDA5-expressing vector or with an empty vector. After 24 h, the cells were harvested and the expression of cMDA5 mRNA was analyzed by RT-qPCR. **(B)** Overexpression of cMDA5 led to a marked activation of the IFN-β promoter in response to poly I:C. WT DF-1 and cMDA5 knockout (KO) cells were co-transfected with the cMDA5-expressing vector or empty vector (400 ng), the cIFN-β-Luc vector (200 ng), and pGL4.53 (20 ng). After 24 h, poly I:C was added for 24 h and IFN-β promoter activity was assessed with a luciferase reporter assay. DF-1 cells were used as a control. **(C)** Overexpression of cMDA5 led to a marked activation of the IFN-β promoter in response to avian influenza virus (AIV). WT DF-1 and cMDA5 KO cells were co-transfected with the cMDA5-expressing vector or the empty vector (400 ng), the cIFN-β-Luc vector (200 ng), and pGL4.53 (20 ng). After 24 h, the cells were infected with AIV (MOI = 1.0) for 8 h and IFN-β promoter activity was assessed with a luciferase reporter assay. DF-1 cells were used as a control. **(D)** WT DF-1 and cMDA5 KO DF-1 cells were infected with PR8-H5N8 influenza virus (MOI = 0.01). After 48 h, viral supernatants were harvested and viral titers measured by the TCID_50_ assay. The data are represented as the mean ± standard deviation (*n* = 3). Data were analyzed using Student’s *t*-test **(A–D)**. *P < 0.05, **P < 0.01, ***P < 0.001, and ****P < 0.0001.

We constructed a cytomegalovirus (CMV) promoter-driven cMDA5 overexpression vector to compensate and rescue cMDA5 in the wild-type (WT) DF-1 and cMDA5 KO clone to better understand its function. After transfecting the cMDA5 overexpression vector into WT DF-1 cells, the expression of cMDA5 mRNA was about 1,000-fold higher than that in cells transfected with the empty vector, indicating that the expression of cMDA5 mRNA was quite low in the absence of PAMPs (Figure 3A). In WT DF-1, the overexpression of cMDA5 induced an approximately 4-fold increase of the IFN-β promoter activity after poly I:C treatment when compared with that in control DF-1 cells transfected with the empty vector. Moreover, the overexpression of cMDA5 in the cMDA5 KO clone induced a significant increase in IFN-β promoter activity (about 280-fold) after poly I:C treatment (Figure 3B).

Next, the IFN-β promoter activity stimulated with AIV (MOI = 1.0) showed a similar tendency to that with poly I:C stimulation in WT DF-1 and the cMDA5 KO clone. In WT DF-1 cells, the overexpression of cMDA5 showed an approximately 4-fold induction of IFN-β promoter activity compared to control DF-1 cells transfected with the empty vector after infection with AIV. The overexpression of cMDA5 in the cMDA5 KO clone significantly induced IFN-β promoter activity (∼50-fold) after stimulation with AIV (Figure 3C).

To further examine whether knockout of cMDA5 affects the replication and growth of AIV, we infected WT DF-1 cells and the cMDA5 KO clone with PR8-H5N8 (MOI = 0.01). After 48 h, we measured the viral titer with the TCID_50_ assay. The results showed that the viral titer in the cMDA5 KO clone was about 30-fold higher than that in WT DF-1 cells, indicating decreased sensing of AIV, thus promoting viral replication (Figure 3D).

### Establishment of cTLR3 KO and MDA5/TLR3 Double Knockout DF-1 Clones

**FIGURE 4:**
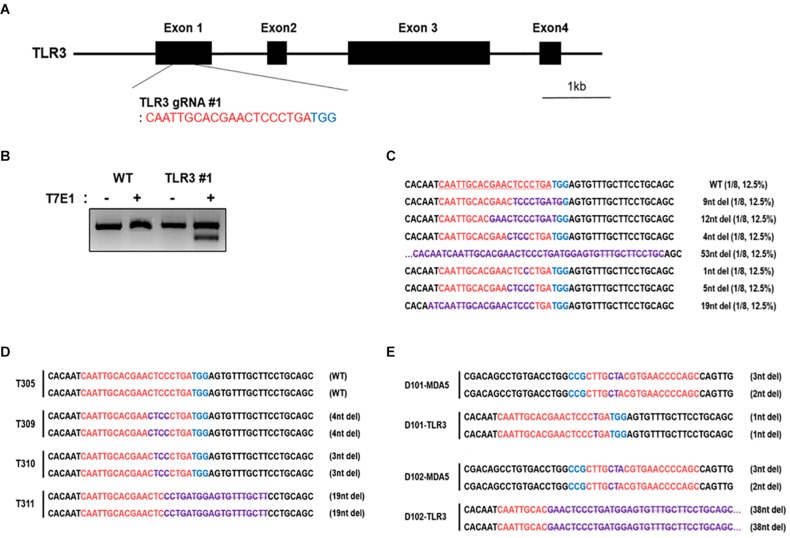
Establishment of chicken TLR3 knockout and MDA5/TLR3 double-knockout cell lines using the CRISPR/Cas9 system. **(A)** Location of cTLR3 gRNA #1 targeting exon 1. **(B)** T7E1 assays were performed in DF-1 cells transfected with the cTLR3 gRNA #1 CRISPR/Cas9 vector. Wild-type (WT) DF-1 cells were used as a control. **(C)** Mutation patterns in TLR3 knockout (KO) DF-1 cells were confirmed by sequence analysis. **(D,E)** Sequence analysis of single TLR3 KO and MDA5/TLR3 double-knockout (DKO) cells after clonal expansion. The *red letters* indicate guide RNA-binding sites, the *blue letters* indicate protospacer adjacent motif (*PAM*) sequences, and. the *purple letters* denote mutation patterns. *T3* denotes single TLR3 knockout cell clones and *D* denotes single MDA5/TLR3 double-knockout cell clones.

To further examine the functional role of cMDA5 and cTLR3 during the recognition of RNA ligands, we established TLR3 KO DF-1 and MDA5/TLR3 double knockout (DKO) DF-1 cell lines using the CRISPR/Cas9 system. First, we constructed cTLR3 gRNA #1 containing the CRISPR/Cas9 vector to target the first exon of cTLR3 (Figure 4A). Transfection of this vector into DF-1 cells resulted in in-del mutations at the target locus, as detected by a T7E1 assay (Figure 4B). To further examine the mutation efficiency of the transfected vector, we examined the target regions of genomic DNA from the transfected cells by DNA sequencing. The sequencing results showed that the mutation rate in cTLR3 #1 was 87.5% (7/8) (Figure 4C). Subsequently, TLR3 KO and MDA5/TLR3 DKO cells were established by expanding a single cell clone of cT#1 (cTLR3 #1)-transfected WT and cMDA5 KO DF-1 cells, respectively. The nucleotide mutations at the target locus of individual TLR3 KO (T305, T309, T310, and T311) and MDA5/TLR3 DKO (D101 and D102) clones were confirmed by DNA sequencing analysis (Figures 4D,E). Finally, we selected T311 and D102 as representative TLR3 KO and first exon-deleted MDA5/TLR3 DKO clones, respectively, for further study.

### Targeted Double Knockout of cMDA5 and cTLR3 Abolished Type I IFN-Mediated Innate Immunity

**FIGURE 5:**
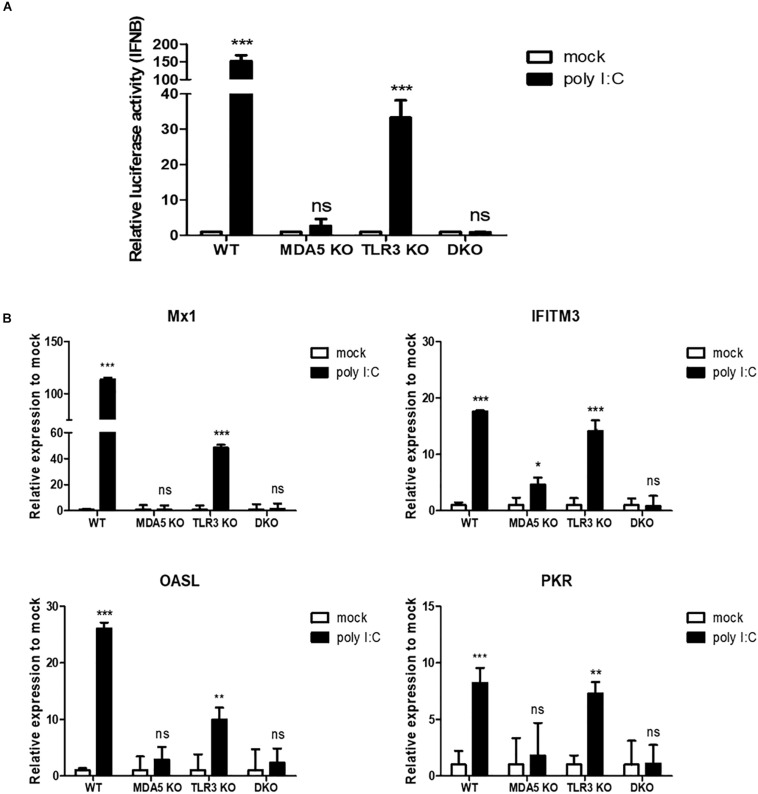
cMDA5 is an indispensable sensor of RNA ligands in chicken cells, whereas cTLR3 serves only a complementary function. **(A)** cMDA5 knockout (KO), cTLR3 KO, and MDA5/TLR3 double knockout (DKO) result in a decreased IFN-β promoter activity in response to poly I:C. Wild type (WT), cMDA5 KO, cTLR3 KO, and MDA5/TLR3 DKO DF-1 cells were co-transfected with the cIFN-β-Luc vector (200 ng) and pGL4.53 (20 ng) and then stimulated with poly I:C for 24 h. After stimulation, the cells were harvested for use in a luciferase reporter assay. **(B)** WT, cMDA5 KO, cTLR3 KO, and MDA5/TLR3 DKO DF-1 cells were stimulated with poly I:C for 24 h. After stimulation, the cells were harvested and the relative expressions of mRNAs encoding interferon-stimulated genes Mx1, IFITM3, OASL, and PKR were assessed by RT-qPCR. The data are represented as the mean ± standard deviation (*n* = 3). Data were analyzed using two-way ANOVA with Bonferroni’s multiple comparison **(A,B)**. *P < 0.05, **P < 0.01, and ***P < 0.001. *ns*, non-significant.

We performed the luciferase reporter assay to analyze IFN-β promoter activity in WT, MDA5 KO, TLR3 KO, and MDA5/TLR3 DKO DF-1 clones in response to poly I:C stimulation. The results showed that IFN-β promoter activity was significantly upregulated in the WT and TLR3 KO DF-1 clones, whereas there was no significant difference in the MDA5 KO and MDA5/TLR3 DKO DF-1 clones (Figure 5A).

Furthermore, we examined representative interferon-stimulated genes [myxovirus resistance protein 1 (Mx1), interferon-induced transmembrane protein 3 (IFITM3), 2′-5′-oligoadenylate synthetase like (OASL), and protein kinase R (PKR)] in WT, MDA5 KO, TLR3 KO, and MDA5/TLR3 DKO DF-1 clones stimulated with poly I:C. The results showed that the expression of antiviral genes was induced significantly in WT DF-1 cells after poly I:C stimulation; however, there was no (or a less significant) difference between mock and poly I:C in the MDA5 KO and MDA5/TLR3 DKO DF-1 clones. The expressions of all the examined antiviral genes in the TLR3 KO DF-1 clone were shown to be significantly upregulated after poly I:C treatment (Figure 5B).

**FIGURE 6:**
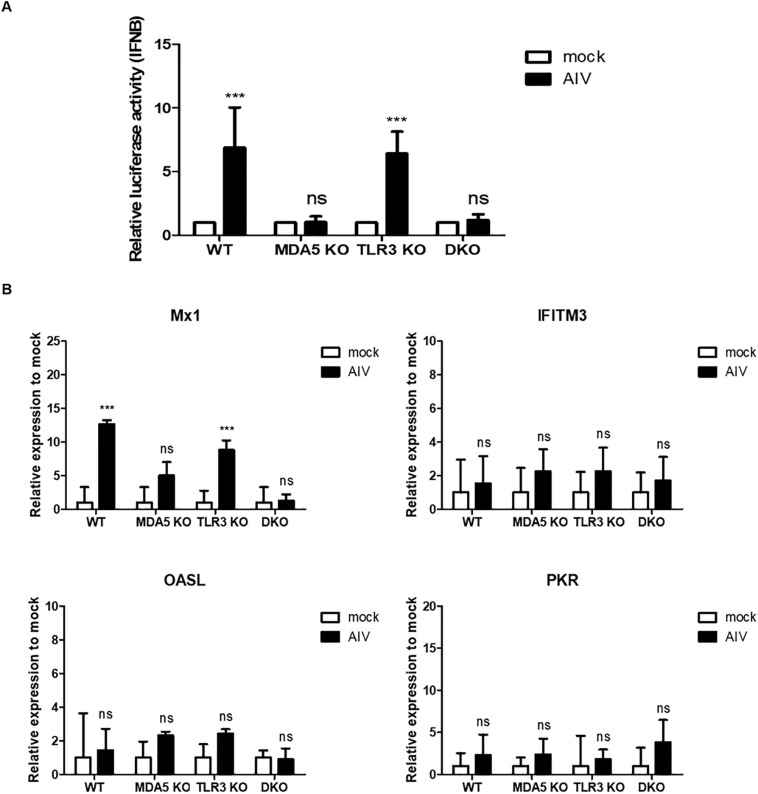
Antiviral response in knockout cell lines stimulated with avian influenza virus (AIV) and NS1 interferes the antiviral genes and IFN signaling. **(A)** cMDA5 knockout (KO) and MDA5/TLR3 double knockout (DKO) failed to induce IFN-β promoter activity in response to AIV. Wild type (WT), cMDA5 KO, cTLR3 KO, and MDA5/TLR3 DKO DF-1 cells were co-transfected with the cIFN-β-Luc vector (200 ng) and pGL4.53 (20 ng) and then infected with AIV (MOI = 1.0) for 8 h. After infection, the cells were harvested for use in a luciferase reporter assay. **(B)** WT, cMDA5 KO, cTLR3 KO, and MDA5/TLR3 DKO DF-1 cells were stimulated with AIV (MOI = 1.0) for 8 h. After stimulation, the cells were harvested and the relative expressions of mRNAs encoding interferon-stimulated genes Mx1, IFITM3, OASL, and PKR were assessed by RT-qPCR. The data are represented as the mean ± standard deviation (*n* = 3). Data were analyzed using two-way ANOVA with Bonferroni’s multiple comparison **(A,B)**. ***P < 0.001. *ns*, non-significant.

Next, we infected WT, MDA5 KO, TLR3 KO, and MDA5/TLR3 DKO DF-1 cells with AIV (MOI = 1.0) and analyzed IFN-β promoter activity. The results showed that stimulation with AIV significantly increased IFN-β promoter activity in the WT and TLR3 KO DF-1 clones, whereas there was no significant induction in the MDA5 KO and MDA5/TLR3 DKO DF-1 clones (Figure 6A).

To investigate antiviral responses, we analyzed the expressions of representative interferon-stimulated genes (Mx1, IFITM3, OASL, and PKR) in WT, MDA5 KO, TLR3 KO, and the MDA5/TLR3 DKO DF-1 clones infected with AIV (MOI = 1.0). The results showed that there was no significant difference between mock and AIV in all cell lines. Only Mx1 showed a significant increase in WT and TLR3 KO DF-1 cells after AIV infection (Figure 6B). Taken together, these results show that MDA5 plays a pivotal role, and that TLR3 plays a minor role, in sensing RNA ligands during innate immune responses in chicken cells.

## Discussion

In most animal species, interferon-mediated innate immune responses against invading pathogens are activated by cellular PRRs. However, chickens lack a RIG-1 homolog, which in mammals plays a major role in sensing RNA ligand PAMPs (31). In chickens, cMDA5 and cTLR (TLR3 and TLR7) act as PRRs that sense RNA ligands (11, 28, 29, 39).

Here, we compared the ability of cMDA5 and cTLR members to sense RNA ligands in chicken cells. First, we assessed the expressions of mRNAs encoding cMDA5, cTLR3, and cTLR7 in DF-1 cells stimulated with poly I:C or AIV to examine which of these receptors is transcriptionally activated. Consistent with previous reports (39, 40), we found that the expressions of mRNAs encoding cMDA5 and cTLR3, but not that encoding cTLR7, increased significantly after stimulation with poly I:C. In addition, the expressions of mRNAs encoding cMDA5, cTLR3, and cTLR7 were significantly upregulated after infection with AIV. The IFN-mediated positive feedback system reported in mammalian species (41, 42) means that all three receptors in chickens can be transcriptionally activated in response to RNA ligands.

To better understand the function of cMDA5, cTLR3, and cTLR7 receptors, we examined the effect of silencing cMDA5, cTLR3, and cTLR7 on the activity of IFN-β in WT DF-1 cells. In mammals, TLR3 plays a pivotal role in inducting interferon through cooperation with RIG-1 (30, 43, 44). Although the induction of IFN-β by AIV was weaker than that after poly I:C stimulation (probably due to viral NS1-mediated inhibition of MDA5 signaling and induction of IFN-β activity) (11, 45), our results agree with those of a previous study showing that silencing of cMDA5 and cTLR3 in WT cells results in a significantly reduced induction of IFN-β activity after stimulation with poly I:C or infection of AIV (39). Here, we employed a luciferase reporter assay rather than the RT-qPCR method to examine IFN-β promoter activity. IFN-β expression is affected by the coordinative activation of several pathways, such as IRF3/IRF7, NF-κB, or MAPK pathways (46–48). NF-κB and MAPK are also involved in the induction of inflammatory cytokines (49, 50). Due to this, it is able to cover important signaling pathways associated with innate immunity through the detection of IFN-β activation (46). To support our data, we showed the expression of interferon-stimulated genes using RT-qPCR together with IFN-β promoter activity.

Next, because cMDA5 plays a dominant role in the recognition of RNA ligands, we used the CRISPR/Cas9 system to establish cMDA5 KO clones. In response to poly I:C and AIV, cMDA5 KO cells appeared to rescue IFN-β promoter activity more efficiently than did WT DF-1 cells, suggesting that knocking out cMDA5 failed to activate the IFN-β promoter. WT DF-1 cells induced IFN-β promoter activity in response to poly I:C or AIV, even without the overexpression of cMDA5, as shown in Figures 1F–G. However, cMDA5 KO clones failed to induce IFN-β promoter activity in response to RNA ligands, causing a huge difference in cMDA5 KO clones upon the overexpression of cMDA5. Furthermore, we found that enforced overexpression of cMDA5 recovered impaired IFN-β promoter activity in the cMDA5 KO clone.

Although previous reports show that the knockdown of cMDA5 in chicken cells did not affect the growth of AIV (33, 39), we found that the growth of AIV in cMDA5-lacking cells was significantly higher than that in WT DF-1 cells. This result indicates that the knockout of cMDA5 makes cells more permissive to virus growth due to the reduced IFN-mediated antiviral activity, in particular the impaired production of IFN-β due to lack of the MDA5 sensing pathway.

Finally, we compared the functional roles of cTLR3 and cMDA5. We established MDA5 KO, TLR3 KO, and MDA5/TLR3 DKO clones by using the CRISPR/Cas9 system. In this study, it was revealed that the IFN-β promoter activity was upregulated in WT DF-1 and TLR3 KO in response to poly I:C, but there was no significant difference in MDA5 KO and MDA5/TLR3 DKO, even after poly I:C stimulation. Furthermore, IFN-β promoter activity stimulated with AIV showed similar results to stimulation with poly I:C. Moreover, we analyzed the expressions of mRNAs encoding interferon-stimulated genes (Mx1, IFITM3, OASL, and PKR) in cell clones exposed to poly I:C (40, 51, 52). Consistent with the results of the IFN-β promoter activity experiments, we found that the expressions of genes involved in the type I IFN pathway were lower in the MDA5 KO and MDA5/TLR3 DKO cells than in WT DF-1 cells; however, this was not the case in TLR3 KO cells, indicating that genes downstream of the type I IFN pathway were not induced in MDA5 KO and MDA5/TLR3 DKO cells due to a reduced or impaired recognition. Even though, we analyzed the expressions of antiviral genes such as the interferon-stimulated genes (ISGs) after stimulation with AIV. It was difficult to detect any induction of gene expression, even in WT DF-1 cells, except Mx1 that showed the highest induction level in response to poly I:C. This phenomenon could be due to the fact that NS1 in influenza virus strongly inhibited IFN signaling; thus, several studies have used an NS1-mutant virus (ΔNS1) to examine antiviral genes (11, 53–55). Therefore, for further analysis, it could be required to examine our four cell lines in response to AIV by using an NS1-mutant virus.

In this study, we suggested that chicken has a different immune system compared with other species and that cMDA5 functions as a pivotal sensor in chicken, as our results reveal from the targeted knockout cell lines. This investigation was performed in an *in vitro* level and it could be used as a platform to further understand the chicken immune system through *in vivo* studies. Moreover, we performed gene knockdown and knockout experiments employing only one cell line, the chicken fibroblast cell line (DF-1), to investigate the chicken immune system. Despite this limitation, DF-1 and HD-11 showed similar tendency using siRNA-mediated knockdown of cMDA5 (11). Although we suggest that similar results will appear in different cells, type I IFN production in response to RNA virus sensing by innate immune sensors was shown to be cell-type specific (56), suggesting that further studies in various cell types must be conducted.

Even though our siRNA-mediated knockdown experiment was highly efficient and showed fairly consistent results, our knockdown experiment showed that cTLR7 was not involved in IFN-β signaling in response to poly I:C or AIV compared to cMDA5 and cTLR3. It speculates that TLR7 may function differently in DF-1 cells or be involved in different signaling pathways. A comparative study between TLRs and RLRs has suggested that non-immune cells, such as epithelial cells and fibroblasts, and also myeloid cells rely on a MAVS-dependent type I IFN production, which means following RLR signaling pathways (56). However, TLR7 is highly expressed in plasmacytoid dendritic cells (pDCs) and B cells (57–59), whereas low expression levels are observed in non-immune cells (60–63). Furthermore, TLR7 is critical to producing type I IFN by pDCs in response to RNA viruses (64). In the case of chicken, previous studies revealed that IFN production by TLR7 appeared to be genotypic-specific (65), and chicken splenocytes could induce IL-1β mRNA, but failed to induce IFN-α/β in response to RNA ligand for TLR7 (66). To clarify the function of TLR7 accurately, further studies are required.

In our data, we used an AIV as a representative RNA virus for the functional assessment of the recognition of RNA ligands by cellular PRRs in the absence of RIG-1 in chicken. A recent study has demonstrated that cSTING acts as a mediator of type I IFN induction through MDA5–STING–IFN-β pathways in response to AIV and Newcastle disease virus (NDV) (67). Furthermore, the knockdown study revealed that cMDA5 is involved in antiviral response to infection of infectious bronchitis virus (IBV) in DF-1 cells (68). It suggests that cMDA5 functions as a sensor of other RNA viruses, such as NDV and IBV. In addition to this, the functional role of cMDA5, cTLR3, or cTLR7 for the recognition of RNA ligands and other RNA viruses beyond AIVs remains to be investigated.

Collectively, these results suggest that MDA5 plays a decisive role in sensing RNA ligands in chicken DF-1 cells, whereas TLR3 plays only a complementary role.

## Conclusion

The ability of host cells to sense invading pathogens is critical for frontline defense against infection. Here, we demonstrate that cMDA5 is a potent sensor of RNA ligands in chicken cells (DF-1), which lack RIG-1. We also show that cTLR3 plays only a complementary role in the sensing of RNA ligands and subsequent regulation of IFN-β responses. However, cTLR7 does not play a significant role in sensing RNA ligands. Collectively, the data suggest that disruption of the critical sensors in host cells results in the loss of ability to recognize pathogens and initiate the expression of antiviral genes.

## Data Availability Statement

The raw data supporting the conclusions of this article will be made available by the authors, without undue reservation, to any qualified researcher.

## Author Contributions

SL, YP, and JH designed the research. SL, KC, and SW performed the experiments. YP and HC interpreted and reviewed the data. SL and YP wrote the first draft of the manuscript. HC, SH, KC, DR, and JH helped to writing the final version of the manuscript.

## Conflict of Interest

The authors declare that the research was conducted in the absence of any commercial or financial relationships that could be construed as a potential conflict of interest.

## Footnotes

1 http://blast.ncbi.nlm.nih.gov
