# Supplementary material for: Targeted Knockout of MDA5 and TLR3 in the DF-1 Chicken Fibroblast Cell Line Impairs Innate Immune Response Against RNA Ligands
Source: Front Immunol. 2020 Apr 30;11:678. doi: 10.3389/fimmu.2020.00678 (PMC7204606; doi:10.3389/fimmu.2020.00678)
Supplement: Supplementary file 1 [file Table_1.DOCX]

**Table S1. List of oligonucleotide sequences and gRNA target sequences used in this study**

| **ID** | **Sequence (5’→3’)** | **Usage** |
| --- | --- | --- |
| MDA5 exon 1-F | GAGACGAGCGCTTCCTCTAC | T7E1 assay and sequencing |
| MDA5 exon 1-R | CCTTATTGCTGGCCCACTGA | T7E1 assay and sequencing |
| TLR3 exon1-F | TGACCGAGTACAGCAATCTG | T7E1 assay and sequencing |
| TLR3 exon1-R | ATCCCCAAAGCCCTGGGAGA | T7E1 assay and sequencing |
| MDA5 qRT-F | CTCTGCGAGAAACCCAACAT | RT-qPCR |
| MDA5 qRT-R | GCCCTCTGCTTCATCTTCAC | RT-qPCR |
| TLR3 qRT-F | TCAGTACATTTGTAACACCCCGCC | RT-qPCR |
| TLR3 qRT-R | GGCGTCATAATCAAACACTCC | RT-qPCR |
| TLR7 qRT-F | TCC ACC CAA CTT ATC TTC AAC GT | RT-qPCR |
| TLR7 qRT-R | ACT CAG CGT CAC CAA TCT CC | RT-qPCR |
| ACTB qRT-F | AGGAGATCACAGCCCTGGCA | RT-qPCR |
| ACTB qRT-R | CAATGGAGG GTCCGG ATTCA | RT-qPCR |
| MDA5 #1 gRNA | AACCGAGGTCGAAACGTACG | gRNA sequence |
| MDA5 #2 gRNA | CGGTGAGCGTGA ACACAAAT | gRNA sequence |
| TLR3 #1 gRNA | AAAGGATCCACTTAGAGCTG | gRNA sequence |
