# Supplementary material for: Targeted Knockout of MDA5 and TLR3 in the DF-1 Chicken Fibroblast Cell Line Impairs Innate Immune Response Against RNA Ligands
Source: Front Immunol. 2020 Apr 30;11:678. doi: 10.3389/fimmu.2020.00678 (PMC7204606; doi:10.3389/fimmu.2020.00678)
Supplement: Supplementary file 2 [file Table_2.docx]

**Table S2. List of siRNA sequences for knockdown experiments**

| **Accession No.** | **Name** | **Direction** | **Sequence ( 5' -> 3' )** | **Start position** |
| --- | --- | --- | --- | --- |
| NM_001193638 | cMDA5-siRNA | Sense | CAGAAGAAGGACUGGUUCU UU | 581 |
|  |  | Anti-sense | AGAACCAGUCCUUCUUCUG UU |  |
| NM_001011691 | cTLR3-siRNA | Sense | GAUUAUGACGCCUAUGUUA UU | 2234 |
|  |  | Anti-sense | UAACAUAGGCGUCAUAAUC UU |  |
| NM_001011688 | cTLR7-siRNA | Sense | GACGUACUUGGACCUCAGU UU | 2431 |
|  |  | Anti-sense | ACUGAGGUCCAAGUACGUC UU |  |
| N/A | Non-specific siRNA^#^ | Sense | CCUACGCCACCAAUUUCGU | N/A |
|  |  | Anti-sense | ACGAAAUUGGUGGCGUAGG |  |

^#^ The non-specific siRNA has no complementary sequence in the chicken genome and was used as a control for gene silencing.
